# Oxidative toxicity in diabetes and Alzheimer’s disease: mechanisms behind ROS/ RNS generation

**DOI:** 10.1186/s12929-017-0379-z

**Published:** 2017-09-19

**Authors:** Waqar Ahmad, Bushra Ijaz, Khadija Shabbiri, Fayyaz Ahmed, Sidra Rehman

**Affiliations:** 1 School of Biological Sciences, University of Queensland, Brisbane, 4072 Australia; 2 Centre of Excellence in Molecular Biology, University of the Punjab, Thokar Niaz Baig, Lahore, 54000 Pakistan; 3 COMSATS Institute of Information Technology Abbottabad, Abbottabad, 22010 Pakistan

**Keywords:** Alzheimer’s disease, Type-2 diabetes mellitus, Oxidative stress, ROS production, Antioxidant treatments, Anti-diabetic drugs

## Abstract

Reactive oxidative species (ROS) toxicity remains an undisputed cause and link between Alzheimer’s disease (AD) and Type-2 Diabetes Mellitus (T2DM). Patients with both AD and T2DM have damaged, oxidized DNA, RNA, protein and lipid products that can be used as possible disease progression markers. Although the oxidative stress has been anticipated as a main cause in promoting both AD and T2DM, multiple pathways could be involved in ROS production. The focus of this review is to summarize the mechanisms involved in ROS production and their possible association with AD and T2DM pathogenesis and progression. We have also highlighted the role of current treatments that can be linked with reduced oxidative stress and damage in AD and T2DM.

## Background

A set of chemical processes through which living bodies sustain their lives called as metabolism. This includes digestion of food, transport into the body cells and excretion of waste materials through well-conserved intermediary metabolism. The metabolic pathways are the bio-chemical processes involving DNA replication, transcription and translation by enzyme catalysed reaction through which food or other chemicals from the body transformed into different chemicals and produce energy for various life functions [1]. In the living organism’s body cells and tissues are always gone through the assembly, and disassembly processes in a regular manner involving several metabolic pathways. Disturbs in metabolic process by any external or internal factors may result in metabolic disorder followed by many types of life-threatening diseases. The understanding of the cellular and molecular mechanism for incurable diseases like Alzheimer’s disease (AD) and Type-2 Diabetes Mellitus (T2DM) has been progressing rapidly, which also enhances the therapeutic approaches [2].

It has been noteworthy that the advancement in diagnostic and therapeutic approaches improved the disease management. However, pathophysiology of many diseases is still under way. AD and T2DM, the two-utmost communal overwhelming diseases caused by neurological and insulin function disorder, have become a major public health concern worldwide [3, 4], and needed to be address effectively. A large-scale clinico-epidemiological data indicates that both T2DM and AD are most common age-associated diseases around the globe. People with T2DM are prone to risk of AD. The first strong evidence regarding the correlation between AD and T2DM was reported in Rotterdam cohort study [3–5]. A number of clinical, epidemiological, biological, molecular and genetic data supports a patho-physiological link between T2DM and AD, including obesity, impaired glucose, cholesterol metabolism, and hypertension [6–8]. Presence of these symptoms altogether known as metabolic syndrome (MetS) and could signify a pathological connection between impaired metabolism and several neurological disorders [9, 10]. Uncontrolled increased blood glucose is a major cause of T2DM, which is associated with injury of insulin-producing pancreatic β-cells or by insulin sensitivity in adipose or muscle tissues [11, 12]. Both T2DM and AD induce disease severity based on same path-physiological mechanisms, including mitochondrial damage and formation of advanced glycation products (AGEs). Both mitochondrial damage and AGEs are influenced by induced oxidative stress, which not only impair mtDNA and RNA but also affect protein and lipids [13, 14]. Several studies found induced levels of DNA, RNA, protein and lipid oxidative products in T2DM and AD like 8-hydroxyguanosine, 8-hydroxydeoxyguanosine, protein carbonyls and nitrotyrosine; and lipid peroxidation markers, for example, 4-hydroxynonenal, F2-isoprostanes, and malondialdehyde [15–21].

Oxidative stress has been proposed to play a significant role in T2DM and AD progression. The present review highlights the complex mechanism involved in the production of reactive oxygen species (ROS), induced oxidative stress, and their impact on T2DM and AD progression. Moreover, we also highlight the possible treatments to cope with the bad effects of oxidative stress in T2DM and AD.

## ROS production and oxidative damage

ROS in living organisms was first described in 1954 [22, 23]. In 1969, theory of oxygen toxicity was expressed in aerobic organisms after the discovery of superoxide dismutase (SOD) by McCord and Fridovich. ROS production can be associated with age-related diseases, their developmental processes and cell singling pathways [24, 25]. Oxidative radicals have very short lifespan and react rapidly with other molecules [26]. Presence of transition metals, especially Fe and Cu can help to clarify and explain oxidative damage to living cells [27]. Important oxidants in the living organism includes ROS, reactive nitrogen species (RNS) and sulphur-centred radicals. Although not all of them are radicals but in many cases, these non-radicals can produce radical species by reacting cellular compounds and damaging them by oxidation [28]. The ROS can be classified into two groups; radicals and non-radicals. The radicals contain superoxide (O^.^_2_^−^), alkoxyl (RO^.^), peroxyl (ROO^.^), hydroxyl (OH^.^), hydroperoxyl (HO^.^_2_) and nitric oxide (NO.). The non-radicals include hydrogen peroxide (H_2_O_2_), organic peroxides (ROOH), aldehydes (HCOR), hydrochlorous acid (HOCL), peroxynitrite (ONOOH/ ONOO^−^), ozone (O_3_) and singlet oxygen (^1^O_2_) [29, 30].

ROS and RNS can be generated through exogenous and endogenous sources [28]. Exogenous sources may include UV radiations (direct oxidation of cellular components) [31, 32], ultrasound, drugs (like narcotics, anaesthetizes, adreamicine, nitroglycerine and belomycinem) [33], food (containing oxidants such as transition metals, aldehydes, fatty acids and peroxides) [34], γ- radiations [35], pollutants, xenobiotics and toxic chemicals (alcohol, phosphine, mustard gas) [36, 37]. The endogenous sources may include neutrophils, cytokines and other components of white blood cells [38, 39], direct ROS producing enzymes such as NO synthase, indirect ROS producing enzymes such as the xanthin oxidase, mitochondrial, metals and side effects of various diseases [40, 41]. These molecules ultimately target the macromolecules like proteins, lipids and nucleotides that result in genome instability and impaired organ functions [30–34]. These molecules are critical for neuronal and pancreatic beta cell stability and functions [42–44]. ROS readily attacks and generates a variety of variety DNA lesions. These lesions could result in DNA base transversions (e.g. G:C to T:A) [35–37]. More than 200 clinical disorders have been associated with early initiation of ROS. These disorders may include T2DM, AD, cardiovascular damage, inflammation, intestinal tract disease, eye diseases, brain degenerative impairments, aging, hemochromatosis, thalassemia, and Wilson disease [45, 46].

**Fig. 1:**
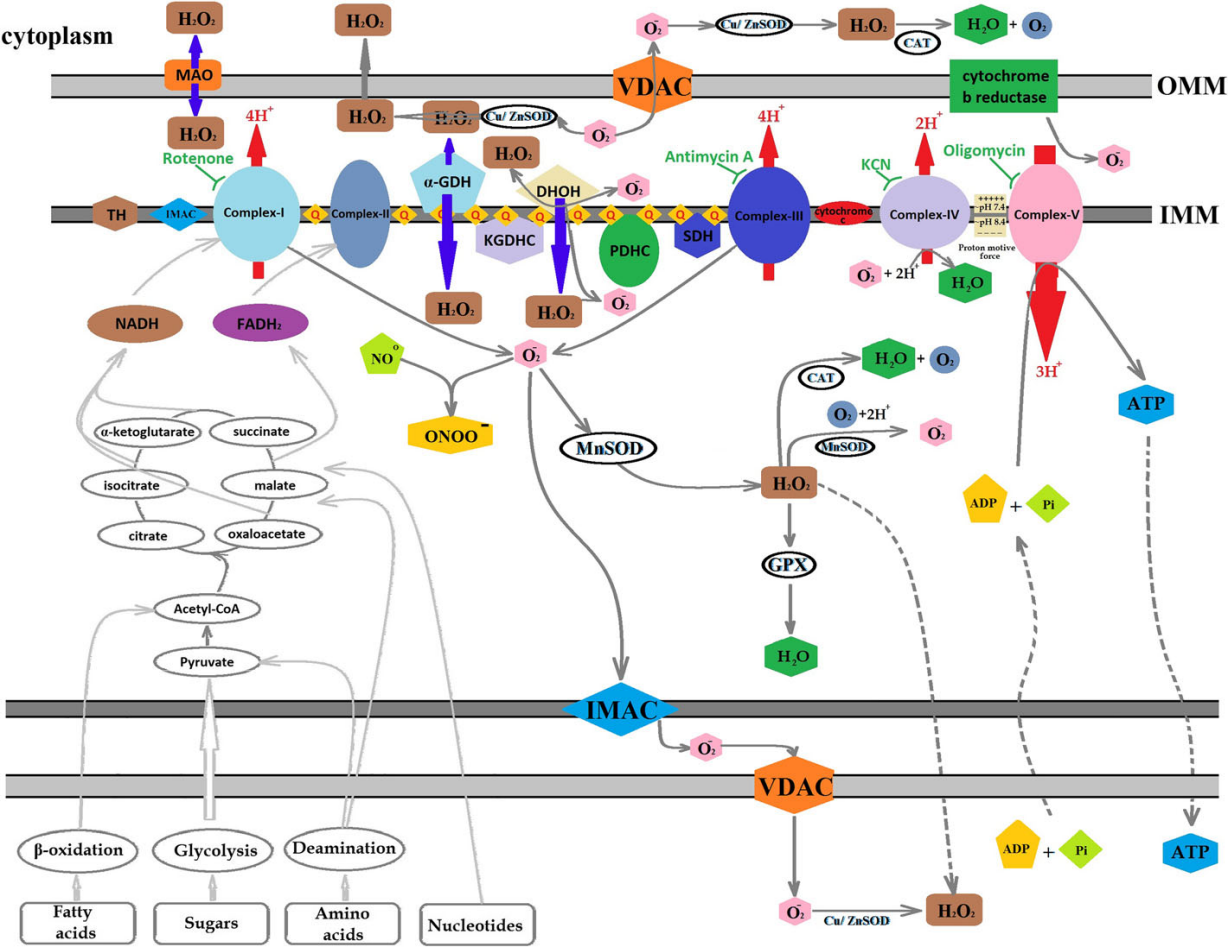
ROS production in mitochondria. Mitochondria is the primary source for ROS production. There are nine different types of enzymes that have the capacity to generate ROS. Among them, some are present on outer mitochondrial membrane (OMM) i.e. Cytochrome b5 reductase and monoamine oxidases (MAO) and while other found in inner membrane, i.e. dihydroorotate dehydrogenase (DHOH), dehydrogenase of α-glycerophosphate (α-GDH), succinate dehydrogenase (SDH), aconitase, α-ketoglutarate dehydrogenase complex (KGDHC), Complex-I and Complex-III. MAO, DHOH and α-GDH produces H_2_O_2_ via direct or indirect biochemical reactions, while cytochrome b5, Complex-I and complex-III produce superoxides. Complex-I produced superoxides in presence of NADH and require tightly bounded ubiquinone. Rotenone can block electron transport by inhibiting ubiquinone and produce ROS, and requires a high degree of redox reduction on the rotenone binding site. The second process involved in ROS production from Complex-I has been known as ‘reverse electron transfer (RET)’. In RET, electrons are transferred against the flow of redox potentials of electron carriers (i.e. from reduced co-enzyme Q to NAD^+^ not to oxygen). Complex-III can produce a lot of superoxides during Q-cycle (a multifarious reaction system involved oxidation of coenzyme Q while, cytochrome c acts as electron carrier/acceptor) that rapidly generate H_2_O_2_ by dismutation. Antimycin can inhibit the quinone reducing site and lead to accumulation of unstable semiquinone and stimulate superoxide production. In the same way KCN and oligomycin can inhibit electron transfer in complex-IV and V respectively, leading to ROS production. SDH is thought to produce ROS via its FAD, while aconitase generate hydroxyl radical by releasing Fe^2+^. PDHC and KGDHC can produce both superoxides as well as hydrogen peroxide. After generation, superoxide can react with many available molecules or free radicals to form different types of free radicals who can accelerate the cellular damage. To cop superoxide, manganese superoxide dismutase (MnSOD) can convert superoxide to hydrogen peroxide that can be additional converted to water and oxygen by the action of several enzymes like catalase (CAT) or glutathione peroxidase (GPX). For further details, see the text

In living organisms, Oxidants and antioxidants play a significant role in regulating free radical balance within the body produced during active metabolism. A disturbed endogenous antioxidant system favors shift towards more pro-oxidants production called as “oxidative stress.” If it shifts towards more production of antioxidants or reducing power termed as “reductive stress” [25, 30, 47–49]. As induced oxidative stress impairs natural defense by unbalancing the oxidants and de-oxidant ratio, balancing oxidative stress is an emerging therapy in various diseases. Figure 1 explains the detailed mechanism involved in the ROS generation in mitochondria.

## ROS as cellular defence

ROS generally maintains the normal physiological functions and cellular defense of the body. Many living organisms survive below a specific homeostatic set point [24]. Although ROS production is beneficial for cellular mechanism, their excessive quantities are always toxic and lead to oxidative damage of many biological functions [25, 50]. To reduce its toxicity, mammalian cells have evolved defense mechanisms, including different DNA base excisions and strand repair enzymes [51, 52]. In this way, living organisms have not only adapted themselves to develop self-protective mechanisms for ROS but also able to use it constructively [24, 53]. Intracellular low level of ROS may act as signaling molecules in many physiological processes, including redox homeostasis and cellular signal transduction [54]. The divergent effects of ROS on many cellular processes suggest that ROS is not merely detrimental by-products, but also generated purposefully to mediate a variety of signaling pathways.

## Oxidative stress in T2DM

DM is a metabolic disorder categorized into two main groups: Type I (Insulin dependent) that is due to immune-mediated beta-cells destruction and lead to insulin deficiency, and Type 2 (Non-Insulin dependent) that is due to insulin secretion defects and resulted in insulin resistance [55]. Prolonged period of high blood-glucose levels generally linked to both macro and micro vascular complications like CVDs, strokes, peripheral vascular diseases, neuropathy, retinopathy and nephropathy [56–58]. In addition to elevated blood-glucose levels, other factors include high-cholesterol level (hyperlipidemia) and oxidative stress leading to high risk of complications [59]. According to epidemiological studies, diabetic mortalities can be explained by an increase in vascular diseases that could be a cause of oxidative damage [60]. Current research reported that apo-lipoprotein component of LDL instead of lipid alone could be a cause of oxidative damage in DM [60].

Production of free radicals and their high levels in diabetic patients could be non-enzymatic (i.e. glycated proteins, glucose oxidation and increased lipid peroxidation) or enzymatic (over/under-expressed levels of enzymes like catalase (CAT), superoxide dismutase (SOD) and glutathione peroxidise (GSH–Px)). These abnormalities may lead to damage of enzymes, cellular machinery and increased insulin resistance due to oxidative stress [61, 62]. Recent studies have provided a clear evidence that the main source of ROS/ RNS production in T2DM is mitochondria [63–65]. Abnormal mitochondrial functions and excessive ROS/RNS production play a primary role in onset T2DM and its complications. These studies also support the possibility for mitochondrial-targeted antioxidant’s therapy of T2DM complications [66].

During cellular metabolism, insulin reacts with it receptors that lead to activation of Akt and translocation of GLUT4 to cell membrane. Impaired oxidative phosphorylation, reduced NADH oxidoreductase and citrate synthase activities resulted in insulin resistance [6, 67]. This insulin resistance could be the result from either impaired fatty acid acetyl-CoA oxidation or from subsequent accumulation of intracellular lipid and diacylglycerol with consequent activation of protein kinase C and ROS production. This impaired fatty acid oxidation resulted in activation of serine kinases followed by phosphorylation of insulin receptor substrates and interfering insulin signal transduction [68].

Multiple studies have observed the presence of oxidative markers like F2-isoprostane and nitrotyrosine in urine, plasma and tissue levels of diabetic patients [69, 70]. ROS and NOS production in DM can be promoted by both enzymatic and non-enzymatic sources. Main enzymatic sources may be endothelial and vascular smooth muscle cells, NADPH oxidase, xanthine oxidase, cyclooxygenase and uncoupled NOS whereas, non-enzymatic sources include mitochondrial respiratory chain, AGES, glucose autoxidation process and activated polyol pathway [71].

ROS production has become a fundamental part in the T2DM pathogenesis and severity [72]. During the normal glucose oxidation process, the final product is NADH and pyruvate. NADH can reduce pyruvate to lactate or donates its reducing equivalents to electron transport chain. On the other hand, in mitochondrial pyruvate enters into Krebs’s cycle, get oxidised and produce CO_2_, H_2_O, NADH and FADH_2_ [73]. In glucose autoxidation, glucose forms radical and converted to reactive ketoaldehydes and superoxide, consequently, produced hydroxyl radical in presence of transition metals via H_2_O_2_ [74, 75]. Superoxide can also form peroxynitrite radicals by reacting with nitric oxide [76, 77]. Hyperglycemia induced superoxide formation in the mitochondrial electron transport chain by driving the inner mitochondrial membrane potential upward through the generation of excessive electron donors in the Krebs’s cycle [78]. This situation resulted in hyperpolarization of mitochondrial membrane potential and increase in ATP/ADP ratio followed by an inhibition of complex-III and electron accumulation at coenzyme Q. Consequently; this situation accelerates free radical formation by partial reduction of O_2_ and reduces ATP synthesis [79, 80].

Superoxide presence decreases glyceraldehyde-3-phosphate dehydrogenase (GAPDH) activity by 66% and resulted in PARP activation and NAD^+^ depletion [81]. In hyperglycemia, glucose conversion to the polyalcohol sorbitol and fructose via the polyol pathway reduces NAD^+^ to NADH. Sorbitol oxidation through NAD^+^ escort to increased cytosolic NADH: NAD^+^ ratio and inhibit the GAPDH activity, and consequently, increased production of triose phosphate [80]. Increased triose phosphate induced formation of methylglyoxal and diacylglycerol (DAG), PKC and PARP activation [82, 83]. Hyperglycemia also increases hexosamine pathway flux because of increased bio-availability of nutrients and enhances fructose-6-phosphate levels by inhibiting GAPDH by ROS [84, 85]. The outcome of the hexosamine pathway is UDP-N-acetyl glucosamine that triggers many transcription factors and pathways, and lead to microvascular complications of T2DM [86, 87].

**Fig. 2:**
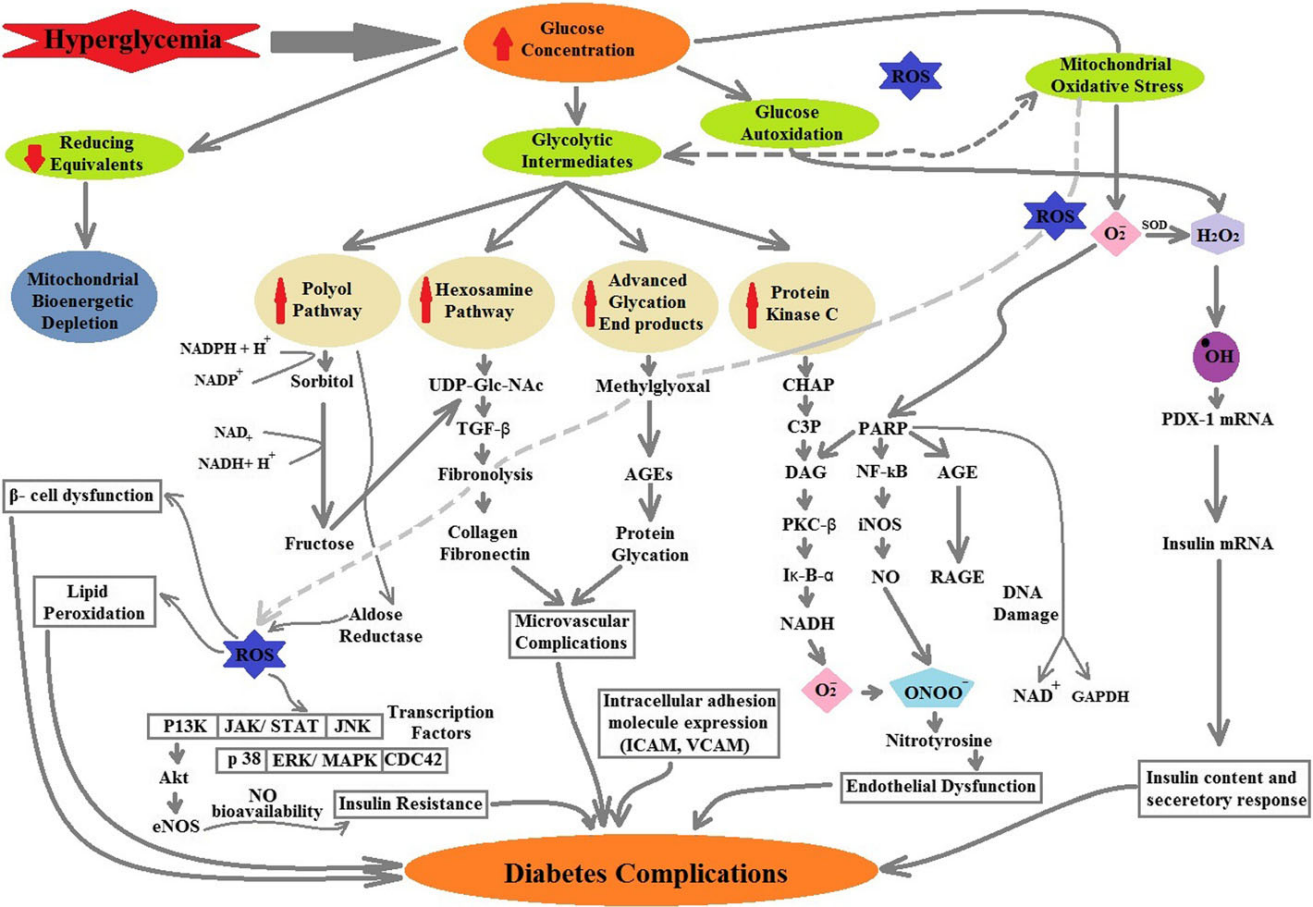
Oxidative stress production and damage in T2DM. Hyperglycemia is considered as major contributor in ROS production and associated- damage in T2DM. Induced glucose concentrations may have led to glucose autoxidation, impaired mitochondrial bioenergetics and over production of ROS. Induced oxidative stress in T2DM can impair a couple of transcription factors and pathways like P13K, JAK/STAT, JNK, p-38, ERK/MAPK and CDC42 that resulted in insulin resistance. The other glycolytic intermediates can have led to microvascular complications and endothelial dysfunctions and prone to several diabetic complications

Overproduction of superoxidase radicals is countered by superoxide dismutase’s (SODs) and by uncoupling proteins (UCPs). In hyperglycemia, over expression of UCPs reduce mitochondrial hyperpolarization and ROS formation, and block the glucose induced cell death. Superoxide radical generation was enhanced in patients with diabetic endothelial cells that promote oxidative stress toxicity [88, 89]. A study by Nishikawa et al. observed the excessive generation of pyruvate via accelerated glycolysis and production of superoxides radicals at the Complex-II level under hyperglycemia [79, 90]. Although glucose is least reactive reducing sugar, it may lead to Amadori product through Schiff base formation by reacting free amino acids. These Aamdori products accumulate on proteins and start the production of AGEs [79, 91] that in turn increase ROS production through binding to RAGE (receptors of AGEs) and resulted in the NF-kB induction and NADPH oxidase formation [92, 93]. NADPH oxidase is major source of O_2_^−^. Levels of NADPH and O_2_^−^ were increased in vascular specimens in diabetic patients [71, 94] and [95]. Binding of AGEs to their receptor RAGE enhanced cytokines and adhesion molecule’s production [96, 97]. This binding also has an abnormal effect on matrix metalloproteinases (MMPs) and transforming growth factor (TGF) [98, 99]. Hyperglycemia also promotes ROS generation by lipid peroxidation of low-density lipoprotein (LDL) [100, 101]. Peroxyl radicals produce hydroperoxides by removing one hydrogen from lipids and propagate further [76]. ROS production also induces cellular stress-sensitive pathways like NF-kB, JNK/ SAPK, P38 MAPK that leads to cellular damage, and late complications in T2DM [102]. Figure 2 summarizes the mechanism involved in progression of T2DM under high oxidative stress conditions.

## Oxidative stress in AD

Clinically AD is characterized by sinister onset, slowly progressive and sporadic disorder, with episodic memory; instrumental signs include aphasia, apraxia, and agnosia, together with general cognitive symptoms, such as impaired judgment, decision-making, and orientation [103]. There are two opinions about the onset of aging. One view is that, it is genetically programmed developmental processes, like the cell senescence, the neuro-endocrine and immunological changes. Another opinion presents that, it is caused by accumulation of somatic mutations and oxidative stress randomly at any time [104]. The crucial events occur during aging progression or their onsets are telomere erosion, oxidative stress and cell senescence. Aged cell phenotype showed futile ROS regulation on mitochondrial super-complexes that causes ROS signalling changes [105]. The neuronal cells are highly sensitive and susceptible to oxidative stress as a result of its high intake of oxygen, lipid content and scantiness of antioxidant enzymes as compared to normal other body tissues [106]. It has been shown that with the passage of time and advance age, ratios of ROS production and antioxidant activities (superoxide dismutase and catalase or glutathione peroxidase enzymes) are disturbed and oxidative damage of macromolecules and their product’s build-up in the brain [107–109].

One of the hallmarks of AD is the accumulation of amyloid beta (Aβ) peptide mostly in mitochondria and it has been shown that Aβ peptide itself can generate ROS in the presence of metal ions such as Fe2^+^ and Cu2^+^ [110]. In mouse models and autopsy analysis of AD patients, mitochondrial dysfunction leads to increased ROS or increased ROS production lead to mitochondrial dysfunction, which in turn enhances Aβ peptide aggregation. Importantly, these elevated markers for oxidative stress precede Aβ deposition and neurofibrillary tangles, suggesting that oxidative stress is an early event involved in AD pathogenesis. Abnormal production of proteins and mtDNA mutation may be due to defective or deficient base excision repair (BER) enzymes and its associated pathways [111–114].

Several hypotheses described oxidative stress as a main culprit in AD pathophysiology [115, 116]. The nervous system is rich source of unsaturated fatty acids and iron. Both these high lipid and iron contents become the targets for oxidative damage in nervous system. In AD pathology, decline in synaptic activities, defects and low energy metabolism with comparatively increased amount of ROS, reduced antioxidants enzymes levels like Cu/Zn-SOD, glutathione (GSH) and catalase in frontal and temporal cortex, and presence of Aβ and NFTs together lead to mitochondrial dysfunctions and neuronal cell death. There are many mechanisms responsible for oxidative stress, like sugar modifications, peroxidation of lipids, oxidation of protein DNA/RNA and production of free radicals by Aβ itself. These molecules are critical for neuronal stability and functions [42–44]. In AD patients, the free-radical production is intimately associated with unique sources of AD pathology. The Aβ (formed by proteolysis of a transmembrane glycoprotein Aβ precursor protein (β-APP)) component of senile plaques is main source of free radical production once it formed outside the neurons via metal-catalysed oxidation of APP [117, 118]. Metals, especially iron plays a significant role in free radical production in AD. Increased iron contents have been found in Aβ and NFTs deposits that catalyses hydrogen peroxide (H_2_O_2_) and form hydroxyl radicals by Fenton reaction. Aβ is also able to boost up the metal ions (such as iron, aluminium and copper) capacity to generate free radicals. Aβ has been shown to produce (H_2_O_2_) and releasing thiobabituric acid reactive substances (TBARS) mainly associated with hydroxyl radicals (OH) via metal ion reduction. Aβ also induce neurodegeneration by targeting microglial NADPH oxidase however, mechanism behind this destruction is poorly understood [119].

AGEs that are present in the senile plaques also produce free radicals by chemical oxidation and degradation, by binding to their receptors (RAGE) or interacting with microglia that surrounds the senile plaques. It results in respiratory blast and production of superoxides and NO [120, 121]. The membranes from the brain are composed of proteins and phospholipids. Presence of aluminium in NFTs stimulates iron-induced lipid peroxidation of oxidisable polyunsaturated fatty acids (PUFAs) that contain weak double bond hydrogen atoms. These PUFAs (like arachidonic acid, docosahexaenoic acid) resulted in multiple aldehydes like acrolein and 4-hydroxy-2-nonenal (HNE). HNE accumulation was shown in NFTs may cause tau phosphorylation, damage or kill primary hippocampus neurons, gene induction, crosslinking of cytoskeletal proteins, cytotoxicity and inhibition of cyclins D1 and D2. HNE also disrupts the binding of histones to DNA and increases chances of DNA oxidation in AD brain [122]. F2-isoprostanes a lipid reliable peroxidation marker is also produced from non-enzymatic peroxidation of arachidonic acid [123].

The oxidation of amino acids like lysine, arginine, proline and histidine via peroxynitrite generates protein carbonyls and nitrile that were increased in AD [124, 125]. Increased levels of protein carbonyls may decrease ATP availability in synaptic terminals and disrupt the cytoskeletal protein assembly [125]. The protein oxidation via nitric oxide produce ONOO radical and nitro-tyrosine that are important non-invasive marker for protein oxidation in AD [125, 126]. The other protein’s oxidation such as ubiquitin, methionine and cysteine is associated with NFTs and the number of tangles has inverse relation with soluble proteins. [127].

The oxidation of DNA and RNA especially mtDNA in AD results in hydroxylated base’s products, DNA-protein crosslinking, strand breakage and impairment of DNA repair system. The levels of 8OHdG were high in AD when compared to the age-matched controls [128, 129]. RNA oxidation is a primary target in AD as RNA is less secure than DNA due to single stranded and specific proteins like histones. The non-coding RNAs are also involved in synapsis, neuronal specification and differentiation, and regulation of dendritic spine development. So their damage due to oxidative stress contributes in development of neurodegenerative diseases specially AD [130, 131]. Nunamara et al., extensively reviewed the RNA oxidation in neurodegenerative diseases and discussed the biological significance and cellular mechanism against RNA oxidation [132].

**Fig. 3:**
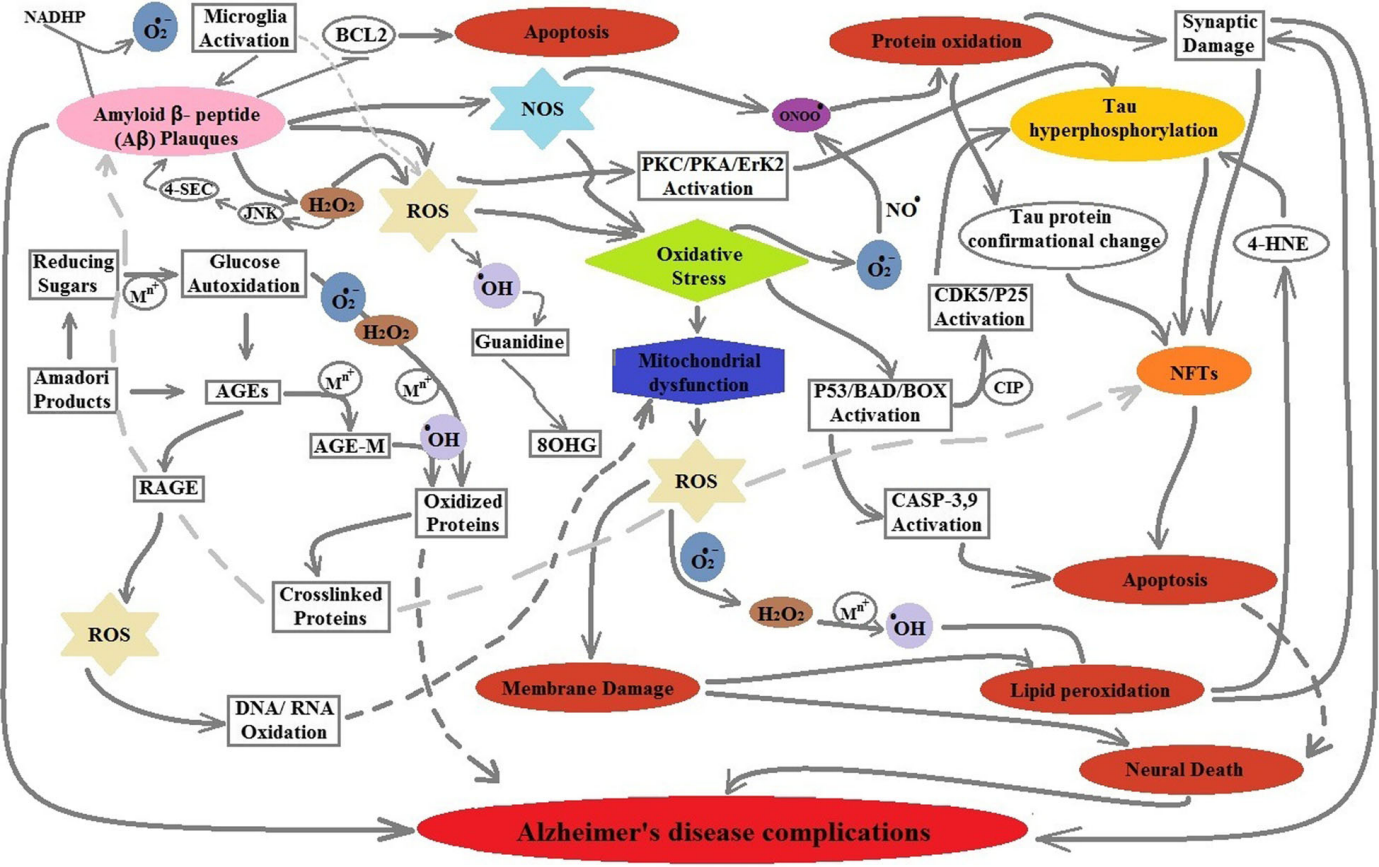
Production and mechanism of oxidative stress in AD. Brain consumes more oxygen than the whole body, and is a rich source of fatty acids and metals that are more susceptible to oxidative damage in AD. Two main hallmarks of AD i.e. Aβ plaques and hyper-phosphorylated tau neurofibrillary tangles (T-NFTs) are involved in production as well as promotion of oxidative damage. Any abnormal increase in ROS due to presence of Aβ and NFTs promote mitochondrial DNA/ RNA damage that resulted in mitochondrial dysfunction and membrane damage. Other damages associated with oxidative stress in AD are autoxidation of glucose that resulted in production of AGES and alternatively induce Aβ- toxicity. As oxidative stress, itself induce Aβ and NFTs formation, the result is induced apoptosis, neuronal death and impaired synapsis

As mitochondria are concerned with a regulatory role in cells through apoptosis, their dysfunction due to oxidative stress may lead a disruption of cellular functions [133, 134]. Apoptosis activates caspases via proteins like BAD, BOX and results in morphological and biochemical changes leading to cell death whereas anti-apoptotic protein BCL-2 over expression may reduce Aβ-induced toxicity in AD via inhibiting p38, MAPK and NFkB pro-apoptotic activation [135–137]. Aβ presence also decreased the mitochondrial respiratory chain complexes activity, while the activity of ATP synthase α-chain reduced with accumulation of NFTs [129, 138, 139]. Figure 3 highlighted the important pathways involved in damage created by oxidative stress in AD.

The effect of oxidative stress on both T2DM and AD remained to define. Intervention to excessive ROS production through scavenging free radicals and increasing antioxidant defence mechanisms are extensively anticipated as anti-aging therapy and also managing AD and T2DM. However, positive and conclusive results have not been achieved even with the association of supplementation and pharmacological or natural compounds. It is possible that few antioxidants may become useless or even harmful sooner or later. Supporting evidence has been obtained from the previous research, which indicates the significant role of oxidative stress in the development of neuronal injury in the diabetic brain and the beneficial effects of antioxidants. We must take into account, that research studies also reported on the failure of antioxidant’s therapies for T2DM. In contrast, the ongoing large clinical trials will also shed additional light on the clinical merit of antioxidant supplementation [66, 140]. These studies suggest that the clearly linking products i.e. deregulated ROS production and oxidative stress in both disorders may lead to common therapy.

## Conclusions

The multi-factorial and inexorable phenomenon of disease complexity of both T2DM and AD leads to gradual reduction of resistance towards oxidative stress, and metabolic disorders that are the major hallmarks of both illnesses. Genetic studies have improved our understanding of pathways that lead to both disorders that highlighting possible interventional targets. Association between AD and T2DM suggests that drug givens to AD patients would be more effective as given to DM [6, 114, 141, 142]. Therefore, targeting T2DM might be more constructive for treating AD. It is also suggested that drugs which used to treat T2DM may affect AD progression ether directly in the brain, provided they pass the blood-brain barrier or indirectly, by modification of systemic blood-glucose concentrations, insulin, inflammatory markers and AGEs. Hence, recent research mostly focuses on treating AD through anti-diabetic drugs that have a direct effect on the brain tissue since brain insulin resistance is often associated with AD [143]. Preclinical and postmortem neuro-pathological studies have identified significant effect of normal insulin signaling in proper functioning through the brain. These findings have given way for investigating novel therapeutic agents for common AD and T2DM pathways [6].

Epidemiological research data has substantiated a strong linkage between T2DM and AD whereas the exact mechanism behind this enhanced risk yet to be discovered. Both AD and T2DM have a high incidence rate at advanced age. Several recent researches reported communal pathological causes between T2DM and AD and therefore, common preventive and therapeutic agents might be effective for both types of disease. The oxidative stress has a transitional part in the AD development. More research is requisite for explore explosive rate in T2DM in the younger generation. Unfortunately, observations made for T2DM and AD drugs seemed to be working in vertebrate and invertebrate models of T2DM, but appears to fail during clinical trials except intranasal insulin therapy. Considering present review, enzyme inhibition is also answering and promising strategy against both types of disease. However, its role in patho-physiology and therapeutics is still needed to explore fully. In conclusion, shared pathogenesis and curative agents make possible to manage life style pattern and use of new therapeutic agents.

## Future perspectives

A better understanding of oxidative stress production and coping in the AD and T2DM might offer some novel targets for therapy. It is further to point out that whether oxidative stress is the eventual basis of pathogenesis; anti-oxidant therapy gets the reward for ultimate treatment. The strategy should be designed in aims of specifically targeting free radical production and oxidative stress that limit its production and progression in the body but how is it possible? Natural products, which are extensively studied to control different diseases by hindering or suppressing ROS production, might be a good choice. Further work is required for better understanding the role of oxidative stress in AD and T2DM progression hence new techniques are compulsory against these substances. Poor knowledge of basic mechanisms involved in aging process, which might interfere to prevent or delay age-related pathologies, like T2DM, cardiovascular disorders, neurodegenerative disorders, and cancer. More investigations are clearly needed to clarify the discrepancy in the role of ROS and antioxidant enzymes in aging process and age-related diseases and to understand the precise role of free radicals play in that processes.

## Abbreviations

AD: Alzheimer’s disease
AGEs: Advanced glycation end products
Aβ: Beta-amyloid
BBB: Blood brain barrier
DAG: Diacylglycerol
GAPDH: Glyceraldehyde-3-phosphate dehydrogenase
GSH: Glutathione peroxidise
GSK3: Glycogen synthase kinase3
H_2_O_2_: hydrogen peroxide
HNE: 4-hydroxy-2-nonenal
KGDHC: α-Ketoglutarate dehydrogenase complex
LDL: Low-density lipoprotein
LMWA: Low molecular weight antioxidants
MAO: Monoamine oxidases
MetS: Metabolic syndrome
MMPs: Matrix metalloproteinases
MPO: Myeloperoxidase
NADH: Nicotinamide dinucleotide
OMM: Outer mitochondrial membrane
RET: Reverse electron transfer
RNS: Reactive nitrogen species
ROS: Reactive oxygen species
SOD: Superoxide dismutase
T2DM: Type-2 diabetes mellitus
TZDs: Thiazolidinediones
α –GDH: α-glycerophosphate
